# Reduction of self-perceived discomforts in critically ill patients in French intensive care units: study protocol for a cluster-randomized controlled trial

**DOI:** 10.1186/s13063-016-1211-x

**Published:** 2016-02-16

**Authors:** Pierre Kalfon, Olivier Mimoz, Anderson Loundou, Marie-Agnès Geantot, Nathalie Revel, Isabelle Villard, Julien Amour, Elie Azoulay, Maïté Garrouste-Orgeas, Claude Martin, Tarek Sharshar, Karine Baumstarck, Pascal Auquier

**Affiliations:** Service de réanimation, Centre Hospitalier Louis Pasteur, Chartres, France; Centre Hospitalier Universitaire (CHU) La Miletrie, Poitiers, France; EA3279 Self-perceived Health Assessment Research Unit Aix-Marseille University, Marseille, France; CHU Dijon Bourgogne, Dijon, France; CHU Hôpital Saint-Roch, Nice, France; CHU Beaujon, Assistance Publique - Hôpitaux de Paris (APHP), Clichy, France; CHU Pitié-Salpêtrière, APHP, Paris, France; CHU Saint-Louis, APHP, Paris, France; Groupe Hospitalier Paris Saint-Joseph, Paris, France; CHU Hôpital Nord, Assistance Publique - Hôpitaux de Marseille, Marseille, France; CHU Hôpital Raymond Poincaré, APHP, Garches, France

**Keywords:** Discomfort assessment, IPREA, Intensive care unit, Critical care, Randomized controlled trial, Cluster

## Abstract

**Background:**

It is now well documented that critically ill patients are exposed to stressful conditions and experience discomforts from multiple sources. Improved identification of the discomforts of patients in intensive care units (ICUs) may have implications for managing their care, including consideration of ethical issues, and may assist clinicians in choosing the most appropriate interventions. The primary objective of this study was to assess the effectiveness of a multicomponent program of discomfort reduction in critically ill patients. The secondary objectives were to assess the sustainability of the impact of the program and the potential seasonality effect.

**Methods/design:**

We conducted a multicenter, cluster-randomized, controlled, single (patient)-blind study involving 34 French adult ICUs. The experimental intervention was a 6-month period during which the multicomponent program was implemented in the ICU and included the following steps: identification of discomforts, immediate feedback to the healthcare team, and implementation of targeted interventions. The control intervention was a 6-month period during which any program was implemented. The primary endpoint was the monthly overall score of self-reported discomfort from the French questionnaire on discomforts in ICU patients (IPREA). The secondary endpoints were the scores of the discomfort items of IPREA. The sample size was 660 individuals to obtain 80 % power to detect a 25 % difference in the overall discomfort score of IPREA between the two groups (design effect: 2.9).

**Discussion:**

The results of this cluster-randomized controlled study are expected to confirm that a multicomponent program of discomfort reduction may be a new strategy in the management of care for critically ill patients.

**Trial registration:**

ClinicalTrials.gov NCT02442934 , registered 11 May 2015.

## Background

It is now well documented that critically ill patients in intensive care units (ICUs) are exposed to stressful conditions and experience discomforts from multiple sources [1–7]. These forms of discomforts, also called stressors, may be differentiated into the following three categories: environmental discomforts (noise, excessive light); discomforts related to the specific organization of ICU care (frequent monitoring, short timeslots for visitors, unavoidable isolation); and discomforts related to the health state of the patient and the care provided (pain, mechanical ventilation or noninvasive ventilation, gastric tube, intravenous access, urinary probe, pleural drain). Some authors have distinguished the discomforts into two other categories: physical (pain, sleep deprivation, thirst, hunger, feeling of cold, feeling of heat) and psychological (communication restriction, lack of autonomy, isolation, anxiety, no respect for intimacy) [3]. These may have significant short-term and long-term consequences for the patients, such as agitation and/or confusion during the ICU stay, which is also called ICU delirium [8–11], or various degrees of anxiety and/or depression [12] or posttraumatic stress disorder after the ICU stay [13–15] that may affect their quality of life [16]. Indirect consequences could also be related to the general representation of the ICU as interpreted by the patients themselves or by their relatives, and patients may develop a fear of the ICU [17, 18]. This could lead to late admissions to the ICU and worsened organ functions, which may impact patient outcomes.

Quantification of discomforts is commonly performed using standardized methods as well as objective or subjective measures. Quantification of noise [19] or lighting [20] and quantification of physiologic parameters [21] were used as stressor indicators, but they did not entirely explore patient discomfort, especially the self-perceived discomfort. Patient-reported outcomes are now considered a better picture of patient feelings and perceptions compared to objective indicators [2, 4, 6, 7] that have led to the development of several ICU-related perceived discomfort tools [3, 4]. Recently, a well-validated questionnaire of self-perceived patient discomforts related to an ICU stay, the IPREA, has been proposed. The IPREA is differentiated from other tools by a validation process based on international guidelines and is performed using a large sample (n = 868) of unselected critically ill patients hospitalized in different types of ICUs (medical, surgical, and mixed medical–surgical) in different institutions (university and non-university hospitals) and a large range (n = 16) of discomforts. Moreover, the IPREA allows the possibility of obtaining a synthetic overall discomfort score [22].

Different actions have previously demonstrated effectiveness in correcting and reducing specific discomforts. The optimization of alarm management and monitoring, the control of the sound level during verbal transmissions between the teams, and the implementation of music sessions, cycling light, and unrestrictive visiting policies have been shown to be efficacious in experimental and/or controlled studies [23–27]. These actions generally focus on one single discomfort, but they have not addressed a more global approach.

An alternative intervention can be proposed. The "assessment-feedback" approach, which combines systematic assessment with feedback to the healthcare teams, previously demonstrated its effectiveness by sensitizing the healthcare team. For example, patient satisfaction assessment combined with feedback has emerged as an important source of information for screening specific problems and developing effective plans of action for quality improvement in healthcare organizations [28]. Also, assessment of patients’ quality of life with systematic feedback to clinicians has been shown to improve patient–physician communication and patient satisfaction in oncology and psychiatry [29–32]. In the specific context of the ICU, we can hypothesize that systematic assessment/feedback of discomforts may also highlight specific discomforts, may have implications for managing care, including considerations of ethical issues, and may assist the healthcare teams in choosing the most appropriate interventions. The identification of specific discomforts may allow for implementing minimal interventions expediently or developing more complex actions. To date, no randomized controlled study has assessed the impact of an "assessment" of discomforts and "feedback" to ICU healthcare teams.

These observations prompted us to establish a multicenter, cluster-randomized, controlled study with the objective of assessing the effectiveness of a multicomponent program of discomfort reduction in critically ill adult patients. This program combines three main components: assessment and identification of discomforts; feedback to the healthcare workers; and implementation of corrective actions. We hypothesized that this program may have a positive effect on the discomfort level self-reported by critically ill patients.

### Objectives

The primary objective was to assess the effectiveness of the multicomponent program of discomfort reduction in the ICU. The secondary objectives were to assess sustainability and a potential seasonality effect of this program.

## Methods/design

### Design

The study design was built to achieve both the primary and the secondary objectives. To achieve the primary objective, a multicenter, cluster-randomized, controlled, single (patient)-blind, two-parallel group study was designed using the recommendations of the Consolidated Standards of Reporting Trials (CONSORT extension for Cluster Trials) statement [33]. The cluster level comprises the ICUs, which are the units of randomization. Patients hospitalized in the ICUs comprise the individual level. All ICUs start with a 1-month period with no intervention, and then they are randomized to one of two groups: a 6-month period during which the multicomponent program (experimental group) is implemented or a 6-month period during which any program (control group) is implemented.

To assess the sustainability of the impact of the program, the study was completed with a second 6-month period during which the program was no longer applied in the ICUs randomized to the experimental group. During this second 6-month period, the program was applied in the ICUs randomized to the control group. We hypothesized that the multicomponent program of discomfort reduction is considered attractive for caregivers in the participating ICUs, leading to an initial preference for the investigators to be randomized to the experimental group. The inferred risk is better adherence to the study in the ICUs randomized to the experimental group in comparison with the other ICUs randomized to the control group and, therefore, an imbalance in the number of included patients in the two groups. That is the reason why the study was designed to offer the opportunity to all the ICUs to implement the program, no matter when the cluster randomization may occur (whether during the first 6-month period or during the second 6-month period).

Moreover, the prolongation of the study for a 6-month period (leading the ICUs that were initially randomized to the control group to test the program) allows assessment of the potential seasonality effect of the program (because the program will be applied during another part of the year) and the ability to obtain eventual confirmation of the impact of the program in other ICUs.

**Fig. 1:**
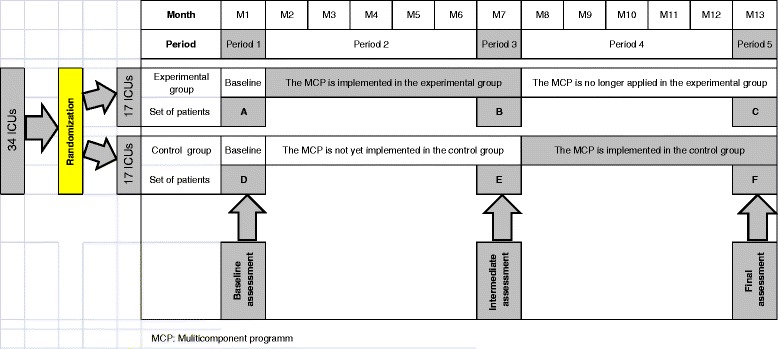
MCP: Multicomponent program

Thus, five periods are differentiated and illustrated in Fig. 1. Period 1 lasts 1 month, during which none of the 34 ICUs apply the multicomponent program. All the eligible patients can be included and can be assessed using the self-perceived discomfort questionnaire, but no feedback of scores will be given to the healthcare teams. Thus, baseline discomfort levels will be available. Period 2 lasts 5 months, during which the 17 ICUs randomized to the experimental group will implement the multicomponent program as detailed below, whereas the 17 ICUs randomized to the control group will not implement it. Period 3 lasts 1 month, during which all the eligible patients of the 34 ICUs can be included and can be assessed using the self-perceived discomfort questionnaire. The 17 ICUs randomized to the experimental group will continue to apply the program with feedback information (last month of the 6-month period for implementation of the program), whereas the 17 ICUs randomized to the control group will receive no feedback. Period 4 lasts 5 months, during which the 17 ICUs initially randomized to the control group will implement the multicomponent program as detailed below, whereas the 17 ICUs initially randomized to the experimental group will no longer implement it. Period 5 lasts 1 month, during which all the eligible patients of the 34 ICUs can be included and can be assessed using the self-perceived discomfort questionnaire. The 17 ICUs randomized to the control group will continue to apply the program with feedback information (last month of the 6-month period for implementation of the program), whereas the 17 ICUs randomized to the experimental group will receive no feedback.

### Partners

Dr. Pierre Kalfon, coordinating investigator, Les Hôpitaux de Chartres (France), is responsible for the study. The methodological support will be provided by the Clinical Research Unit (*Unité Aide Méthodologique à la Recherche Clinique*, AP-HM, France) and the Self-Perceived Health Assessment Research Unit (Aix-Marseille University, Marseille, France). A steering committee includes the coordinating investigator, six French ICU experts, and three nursing managers, each of them working in one participating ICU center, and the head of the Clinical Research Unit (see Table 1 for composition and affiliations of the members of the steering committee). This committee will have the role of elaborating a set of recommendations to prevent each discomfort explored by the 16-item questionnaire. This set of recommendations consists of short sentences that may be considered a minimal charter for reducing the main discomforts perceived by the patients hospitalized in the ICU. This committee can also control the proper conduct of the trial in terms of methodological, ethical, and logistical aspects.

**Table 1:** Coordinating investigators, co-investigators, healthcare workers, methodology and biostatistics team, and steering committee

	Hospitals (all in France)
*Coordinating investigator*	
Dr. Pierre Kalfon	Hôpital Louis Pasteur, CH de Chartres, Chartres
*Co-investigators (alphabetical order)*	
Pr. Julien Amour	CHU Pitié-Salpêtrière, AP-HP, Paris
Pr. Elie Azoulay	CHU Saint-Louis, AP-HP, Paris
Dr. Audrey Berric	Hôpital Sainte-Musse, CH intercommunal de Toulon – La Seyne-sur-mer, Toulon
Dr. Claire Boulle-Geronimi	CH de Douai, Douai
Dr. Olivier Collange	CHU Hôpital Civil, Strasbourg
Dr. Philippe Estagnasie	Clinique Ambroise Paré, Neuilly-sur-Seine
Dr. Bernard Floccard	CHU Edouard Herriot, Lyon
Dr. Arnaud Follin	CHU Hôpital Européen Georges Pompidou (HEGP), AP-HP, Paris
Dr. Maité Garrouste-Orgeas	Groupe Hospitalier Paris Saint-Joseph, Paris
Pr. Carole Ichai	CHU Saint-Roch, Nice
Dr. Quentin Levrat	Groupe Hospitalier de La Rochelle - Ré – Aunis, La Rochelle
Pr. Claude Martin	CHU Hôpital Nord, AP-HM, Marseille
Pr. Olivier Mimoz	CHU La Milétrie, Poitiers
Dr. Martine Nyunga	Hôpital Victor Provo, CH de Roubaix, Roubaix,
Pr. Laurent Papazian	CHU Hôpital Nord, AP-HM, Marseille
Dr. René-Gilles Patrigeon	CH d’Auxerre, Auxerre
Pr. Catherine Paugam	CHU Beaujon, AP-HP, Clichy
Pr. Julien Pottecher	CHU Hautepierre, Strasbourg
Pr. Jean-Pierre Quenot	CHU Dijon Bourgogne, Dijon
Dr. Anne Renaud	CHU Hôpital de la Cavale Blanche, Brest
Pr. René Robert	CHU La Milétrie, Poitiers
Dr. Thomas Signouret	Hôpital Européen de Marseille (HEM), Marseille
Dr. Georges Simon	CH de Troyes, Troyes
Dr. Achille Sossou	CH Emile Roux, Le Puy en Velay
Pr. Tarek Sharshar	CHU Raymond Poincaré, AP-HP, Garches
Dr. Antoine Tesniere	CHU Cochin, AP-HP, Paris
Dr. Didier Thévenin	CH de Lens, Lens
Dr. Marion Venot Dr. Coralie Vigne	CHU Saint-Louis, AP-HP, Paris CHU Hôpital Nord, AP-HM, Marseille
*Healthcare workers*	
Mrs. Marie-Agnès Geantot	Chief nurse, CHU Dijon Bourgogne, Dijon
Mrs. Nathalie Revel	Chief nurse, CHU Saint-Roch, Nice
Mrs. Isabelle Villard	Chief nurse, CHU Beaujon, AP-HP, Clichy
*Methodology and biostatistics team*	
Pr. Pascal Auquier	EA3279 Self-perceived Health Assessment Research Unit Aix-Marseille University, Marseille, France and Department of Epidemiology and Health Economy, AP-HM, Marseille
Dr. Karine Baumstarck	
Mr. Anderson Loundou	
*Steering committee*	
Dr. Pierre Kalfon (chair)	Hôpital Louis Pasteur, CH de Chartres, Chartres
Pr. Pascal Auquier	EA3279 Self-perceived Health Assessment Research Unit Aix-Marseille University, Marseille, France and Department of Epidemiology and Health Economy, AP-HM, Marseille
Pr. Olivier Mimoz	CHU La Milétrie, Poitiers
Mrs. Marie-Agnès Geantot	Chief nurse, CHU Dijon Bourgogne, Dijon
Mrs. Nathalie Revel	Chief nurse, CHU Saint-Roch, Nice
Mrs. Isabelle Villard	Chief nurse, CHU Beaujon, AP-HP, Clichy
Pr. Julien Amour	CHU Pitié-Salpêtrière, AP-HP, Paris
Pr. Elie Azoulay	CHU Saint-Louis, AP-HP, Paris
Dr. Maité Garrouste-Orgeas	Groupe Hospitalier Paris Saint-Joseph, Paris
Pr. Claude Martin	CHU Hôpital Nord, AP-HM, Marseille
Pr. Tarek Sharshar	CHU Raymond Poincaré, AP-HP, Garches

*Note*: CH = Centre Hospitalier (community hospital); CHU = Centre Hospitalier Universitaire (academic tertiary care hospital); AP-HP = Assistance Publique - Hôpitaux de Paris; AP-HM = Assistance Publique - Hôpitaux de Marseille

The study will be performed in 34 French adult ICUs under the supervision of 29 co-investigators (Table 1). This work is supported by institutional grants from the French 2012 *Programme Hospitalier de Recherche Clinique National*.

### Participants

The participating ICUs will comprise the cluster level and the patients managed in the participating ICUs will comprise the individual level. Inclusion and exclusion criteria are provided for each level unit. The details of the inclusion and exclusion criteria of clusters and individuals are provided in Table 2 and Table 3, respectively.

**Table 2:** Selection criteria of ICUs

*Inclusion criteria*	- Adult ICU - Medical, surgical, or medical–surgical ICU - Agreement of the chief responsible - Willingness to be randomly allocated to one of the two groups - Presence of a voluntary physician –nurse pair - No planned interventions that may affect discomfort during the study period
*Exclusion criteria*	- ICU with an expected recruitment ratio (number of collected questionnaires/number of eligible patients) <0.6

**Table 3:** Selection criteria of patients

*Inclusion criteria*	- Adult patients (≥18 years of age) of either sex - Subject discharged from one of the included ICUs during one of the 1-month periods P1, P3, or P5) (Fig. 1) - Subject with ICU stay of 3 calendar days or more - Subject alive on ICU discharge day - Subject consenting to participate in the study
*Exclusion criteria*	- Minors - Subject under trusteeship - Subject deceased during the ICU stay - Subject with ICU stay of 2 calendar days or less - Subject transferred to another ICU while mechanically ventilated - Subject with emergency discharge - Subject with diminished cognitive capacity based on the investigator’s opinion - Subject not consenting to participate in the study - Subject misunderstanding the written and spoken French language

#### Eligibility criteria for ICUs

Eligible ICUs may have the following main criteria: adult ICU; presence of a voluntary physician–nurse pair who accept the role of local champions to reduce patient discomforts and coordinate the whole program based on targeted interventions; and no planned implementation of other interventions that may affect discomfort during the study period.

#### Eligibility criteria for patients

The main inclusion criteria are the following: age 18 years or older; admitted to one of the included ICUs during the predetermined study period with ICU stay of 3 calendar days or more; and alive on the day of ICU discharge. The main exclusion criteria are the following: deceased patient during the ICU stay; ICU stay of 2 calendar days or less; patient younger than age 18 years; patient under trusteeship; patient refusing to participate in the study; patient with diminished mental capacity; patient not understanding French sufficiently to be questioned; patient transferred to another ICU while mechanically ventilated; and emergency discharge. In accordance with French law, oral consent will be obtained from each participant after delivery of the study objectives and implications of the potential participation.

### Experimental and control interventions

#### Experimental intervention: the multicomponent program

During a 6-month period, the ICUs randomized to this group will apply the multicomponent program. Before the beginning of the program, the following proceedings will be achieved. Guidelines will be elaborated by the steering committee containing concise recommendations to prevent each discomfort item that should be used as reminder messages to healthcare teams. In each ICU, two local champions, an ICU physician involved in the continuity of care and the ICU managing nurse or another experimented nurse to substitute the ICU managing nurse in case of absence, are identified before the beginning of the program. These two people will be in charge of the local coordination of the program. Each participating ICU will be supplied with tablets with Internet connection unless each room in the ICU is equipped with a computer and Internet access. To optimize the inclusion of potentially eligible patients, the bedside nurse will have to enter patient demographic data in an anonymized form starting from the day of ICU admission (prescreening process). On the day of ICU discharge, the bedside nurse will find the patient, whose information has already been entered; the nurse will connect to the electronic-specific database and will test eligibility and exclusion criteria by following a step-by-step approach (screening process). If the patient presents eligibility criteria and has no exclusion criteria, then the bedside nurse will have to assess the discomforts perceived by the patient. To ensure adequate training of the nursing staff, the application (access to the specific website and use of a preproduction database) will be used in each ICU for a training period of several weeks with technical and educational support provided by the coordinating investigator and the coordination team before the first patient is included.

The program comprises the following three stages:

1. Discomforts assessment: a web-based systematic measurement of the patient discomforts will be performed using IPREA by the bedside/institutional caregiver (including nurses and assistant nurses) in charge of the patient (presented in Table 4). On the day of ICU discharge, the nurse (or the assistant nurse) will ask patients to rate the severity of each discomfort experienced during the entire stay in the ICU (not just during the last day in the ICU) using a 10-point numerical rating scale. Each discomfort is scored on a scale from 0 to 10 (example: 0 = no pain and 10 = worst pain possible). A random process will allow the nurse to ask the patient in a random order to reduce contamination between items.
2. Feedback of discomfort scores: two kinds of feedback will be organized. First, immediate feedback corresponding to the three discomforts reported with the highest scores by the patient will be forwarded to the bedside/institutional caregiver. Concise messages used as reminders (from the set of recommendations previously elaborated by the steering committee) will be displayed on the screen before the application is closed by the nurse or the assistant nurse. Moreover, the overall discomfort score will be automatically calculated and presented on the screen to the nurse at the end of the administration of IPREA. Second, monthly and cumulative discomfort scores and their relative ranking compared to other ICUs assigned to the experimental group will be forwarded each month to the local champions by the coordination team.
3. Targeted interventions: an implementation of targeted interventions based on the monthly feedback will be planned in each ICU to reduce discomforts. Once the local champions have received the results of their ICUs, they will organize a meeting with their ICU team to present and comment on their results. At the end of these monthly meetings, the local champions will consult with the staff (physicians, nurses and assistant nurses, psychologists if regularly present in the ICU) and select appropriate actions. These actions may correspond to the most important perceived discomforts in the ICU, those perceived at a higher level in comparison with the other ICUs randomized in the experimental group, or those that appear most easily preventable. During these meetings, local champions will also assess the effectiveness of previously decided measures, identify any barriers to implementation, and suggest solutions to resolve these barriers. It is important to note that the measures implemented in the ICU are not decided by the steering committee or by the coordination team. The local champions consult with their staff to define the best targeted measures for their ICUs. Therefore, these measures will be different from one ICU to another. After each monthly meeting, local champions will send the meeting report with the list of participants to the coordination team. Beyond this role of meeting moderators, the local champions will raise the awareness of the staff about discomfort prevention and disseminate and facilitate the ongoing measures for discomfort prevention on a daily basis.

**Table 4:** The French IPREA questionnaire for assessing self-reported discomforts perceived by the critically ill patients, original version

1	Avez-vous souffert du ***bruit*** (alarmes, radios, sonneries de téléphone, conversations) de jour comme de nuit?
2	Avez-vous souffert de la ***lumière*** (éclairage trop important dans la chambre ou dans le couloir surtout la nuit?
3	Avez-vous souffert du ***lit*** (matelas trop dur ou trop mou, matelas à eau, tête de lit trop ou pas assez relevée, lit trop bas ou trop haut, barrières, mauvais oreillers, etc.)?
4	Avez-vous souffert du manque de ***sommeil*** par rapport à d'habitude?
5	Avez-vous souffert de la ***soif***?
6	Avez-vous souffert de la ***faim***?
7	Avez-vous souffert du ***froid***?
8	Avez-vous souffert de la ***chaleur***?
9	Avez-vous eu des ***douleurs***, même si elles étaient présentes avant l'hospitalisation, y compris les douleurs liées aux piqûres ou lors des changes ou de la toilette matinale?
10	Avez-vous souffert d'être entouré de ***tuyaux*** (pour les perfusions, les connections des électrodes fixées sur le thorax, l'oxygène dans le nez ou sur le masque, la pince pour surveiller l'oxygénation, etc.)?
11	Avez-vous été gêné par le fait que votre ***intimité*** ne soit pas suffisamment respectée (par ex. pendant la toilette matinale, les changes, l'examen par les médecins, ou les visites médicales)?
12	Avez-vous souffert d'***angoisse*** (peur parfois panique par exemple qu'un appareil important fonctionne mal, provoquée parfois par le déclenchement d’alarmes sonores) ou vous êtes vous senti très anxieux durant votre hospitalisation?
13	Avez-vous souffert d’***isolement*** (être seul dans votre chambre, parfois sans voir d'infirmiers ou de médecins à proximité) et sans entendre de bruit?
14	Avez-vous été gêné par la limitation des ***visites*** des membres de votre famille ou de vos amis selon les horaires de visite en vigueur dans le service?
15	Avez-vous été gêné de ne pas avoir de ***téléphone*** dans la chambre?
16	Avez-vous été gêné de n'être pas assez ***informé*** de votre état ou de ce qu'on allait vous faire, de l'évolution de votre maladie, de votre date de sortie de réanimation, des suites, que ce soit par les infirmières ou les médecins?Note: each discomfort item is in bold

#### Control intervention: usual care management

During a 6-month period, the ICUs will apply any specific program. The control period will be based on usual care management. However, during the last month of this 6-month period, the caregivers will have to assess discomfort among the eligible patients without receiving any type of feedback.

#### Randomization of clusters

Active consultation is conducted by the coordinating investigator among ICU heads in each potential participating ICU to ensure agreement regarding implementation of the program on the ICU level. Computer-generated randomized lists will be drawn up before the beginning of the study using a permuted block design, for which the clinical research unit (AP-HM) will be responsible. The randomization will be stratified by the average monthly number of patients not deceased during ICU discharge during the year preceding the start of the study (three levels were considered: ≤35, 36–59, and ≥60). The randomization will assign each ICU (1:1 allocation ratio) to either control or experiment. The investigators and caregivers will not be blinded, but all participants will be unaware of the ICU allocation.

### Data collection

The following ICU-specific parameters will be recorded: type of ICU (medical, surgical, or mixed medical–surgical); number of beds; mean of stay duration; annual mortality rate.

Data related to the patient will be recorded from an electronic case report form (eCRF) specifically elaborated for the study. The following data will be collected: demographics (sex, age); health status before the ICU stay using the Knaus score [34] and the McCabe index [35]; health status at ICU admission using the Simplified Acute Physiology Score (SAPS II) [36]; type of admission (medical, scheduled surgical, or emergency surgical); health status from the ICU admission to ICU discharge (use and duration in days of mechanical ventilation, noninvasive ventilation, administration of vasopressors, renal replacement therapy, type and number of invasive procedures, sedation duration, delay between interruption of the sedation and discharge from the ICU); and self-perceived discomforts (IPREA, see the Endpoints section) assessed on the day of ICU discharge during the specific periods of assessment.

### Endpoints

The endpoints pertain to the individual level. Patient discomfort will be assessed using the French self-reported discomfort IPREA questionnaire ("Inconforts des Patients de REAnimation": Discomforts perceived by ICU patients) [22]. IPREA is a well-validated specific questionnaire that is elaborated using standard procedures. This self-reported questionnaire includes 16 discomfort items: noise; excess of light; discomfort related to sleeping in a bed different from the one the patient has at home; sleep deprivation; thirst; hunger; feeling of cold; feeling of heat; pain; being tied down by perfusion lines, tubes, or as a result of connections due to monitoring devices; no respect for intimacy; anxiety; isolation; limited visiting hours; absence of phone; and lack of information. Each item is scored from 0 (minimal discomfort) to 10 (maximal discomfort). The overall discomfort score is calculated as the mean of all the scores reported for each discomfort item multiplied by 10, yielding an overall discomfort score between 0 (minimal overall discomfort) and 100 (maximal overall discomfort). The questionnaire is administered on the day of ICU discharge. The time frame considers the period from the date of admission to the ICU until the day of discharge from the ICU.

### Primary endpoint

The primary endpoint is the monthly overall discomfort score of the 16-item IPREA (mean of all the overall discomfort scores available during the month).

#### Secondary endpoints

The secondary endpoints are the monthly discomfort scores of the 16 items included in IPREA.

### Statistical considerations

#### Sample size, power, and statistical methods

The sample size was determined to detect a 25 % difference in the overall score of IPREA between the two groups. This 25 % difference, considered to be clinically significant, was based on a previous report indicating a mean overall discomfort IPREA score of 22 (standard deviation of 14). The intracluster correlation coefficient (ICC) was estimated from the database of the IPREA questionnaire validation study. With a design effect calculated to be 2.9 (ICC = 0.1; expected equal cluster sizes = 20), a two-sided significance level of 5 %, and 80 % power, 630 IPREA scores are needed (315 in each group) to detect this difference. To prevent 10 % of the questionnaires being unusable or invalid, a total of 700 individuals should be included.

#### Data analysis

The data will be analyzed using SPSS version 17.0 software and SAS/STAT® version 9.2.

The primary (overall score of IPREA) and secondary (16-item scores of IPREA) endpoints will be analyzed on the individual patient level using cluster level summaries, which will account for the correlation between patients in the same center. Analysis methods based on individual patient-level data perform well when the number of clusters is high [37]. The overall score of discomfort and the scores for each item will be compared between control and experimental groups. No interim analysis is planned.

The primary analysis will be performed on the intention-to-treat population corresponding to all included individuals in each participating ICU. A secondary analysis will be performed on the per-protocol population corresponding to all included individuals who complete the IPREA questionnaire. Supplementary sensitivity analyses will be performed on a subgroup of ICUs corresponding to ICUs with a recruitment ratio (ratio of number of collected questionnaires to eligible patients) higher than 0.7, 0.8, and 0.9.

Descriptive analyses will provide ICU and patient characteristics for each group (control and experimental groups). The normality of the parameters will be estimated using frequency histograms and the Shapiro test. Comparisons between the two groups will be performed using the main individual characteristics using chi-squared tests or Fisher exact tests for qualitative variables and Student *t*-tests or Mann–Whitney tests for continuous variables.

All analyses will account for clustering to ensure correct type I error rates and confidence intervals [38]. Appropriate statistical methods to account for clustering between patients in the same cluster will be performed using linear mixed-effects models or generalized estimating equations with inclusion of random effects for individual ICUs and assessment of possible confounding.

The primary endpoint (mean of the overall discomfort scores collected during the 1-month period, B versus E in Fig. 1) will be analyzed without adjustment by comparison of IPREA scores between the two groups using Student *t*-tests or Mann–Whitney tests according to the variable distribution. A set of secondary analyses will be adjusted for covariates of clinical interest using generalized linear mixed regression models including the group (interventional/control), ICUs and individuals (as random variables), a priori covariates (patient age, gender, ICU stay duration, mechanical ventilation duration, and type of admission), and other co-variables selected with a threshold *p*-value <0.05 during univariate analysis. These models will be performed using the MIXED SAS procedure (version 9.2). The same procedure will be performed on the 16 IPREA scores for each discomfort item (B versus E in Fig. 1).

Generalized linear mixed regression models will be performed to assess the sustainability of the effect by comparison of monthly means of overall discomfort scores collected during periods P3 and P5, respectively, reported by patients included in the experimental group at 6-month intervals (B versus C in Fig. 1) and the seasonality effect by comparison of monthly means of overall discomfort scores collected during periods P1 and P3, respectively, reported by patients included in the control group at 6-month intervals (D versus E in Fig. 1).

### Ethical aspects, laws, and regulations

The study will be conducted in accordance with the Helsinki Declaration, French laws and regulations (*Code de la Santé Publique, article L.1121-1/Loi de Santé Publique no. 2004–806 du 9 août 2004 relative à la politique de santé publique et ses décrets d’application du 27 août 2006*), and the International Conference on Harmonization (ICH) E6 Guideline for Good Clinical Practice. Regulatory monitoring will be performed in accordance with the French law requiring the approval of the French ethics committee (*Comité de Protection des Personnes Tours Région Centre-Ouest 1*, 28 August 2013, reference number 2013-S10). All records and subjects’ identities will remain confidential in accordance with the following French regulations: the French National Committee of Informatics and Liberties (*Commission nationale de l’informatique et des libertés*, 20 March 2014, reference number DR-2014-097) and the French Consultative Committee for Data Processing in Health Research (*Comité consultatif sur le traitement de l’information en matière de recherche dans le domaine de la santé*, 12 December 2013, reference number 13.642bis). Oral consent will be collected for each participant.

## Discussion

To our knowledge, this is the first study assessing the effectiveness of a program of discomfort reduction for ICU patients using a randomized controlled design, which is the most appropriate design to demonstrate the efficacy of a new experimental intervention in accordance with the levels of evidence classification of the Evidence-Based Medicine Working Group (Evidence-Based Medicine Working Group 1992; produced by Bob Phillips, Chris Ball, Dave Sackett, Doug Badenoch, Sharon Straus, Brian Haynes, and Martin Dawes since November 1998; updated by Jeremy Howick in March 2009).

We hypothesize that routine assessment of patient discomforts will be useful for further investigation and implementation of targeted preventive/curative actions. A previous study using the IPREA questionnaire for patient discomforts assessment has shown that most of the identified discomforts may be relatively easily relieved by interventions conducted by the ICU team. This program is in accord with the message of the 2009 French consensus conference centered on “a better life in ICU,” which lists recommendations to reduce stressors and discomforts. This program is based on the "plan-do-check-act" (PDCA), which is a management method for the control and continuous improvement of widespread processes and products in business and contributes to quality management and continuous quality improvement in healthcare [39]. The PDCA involves the planning and selection of interventions intended to improve the selected process, implementation of the selected intervention, assessment of its effectiveness, and embedding of changes into the existing quality improvement structure. If this program is effective, then it may provide a significant improvement in the negative experiences reported by ICU patients, such as stressors and discomforts.

However, some issues related to this specific protocol study should be developed. First, the choice of a cluster design should be discussed. Because the experimental program is applied to the whole ICU and affects all the individuals within the ICU, the individual randomization will not be appropriate [40]. To fit the intervention application and to avoid contamination within the unit, cluster randomization is advocated [41, 42]. However, one bias should also be taken into account: because of the consecutive inclusion of patients, there is a greater chance of fluctuations, potentially causing selection bias, which needs to be assessed and adjusted for if present. The cluster approach has previously addressed pertinent issues of ethics. The decision regarding whether a particular cluster will participate in the trial is made by one single person (also called a guardian in a previous debate article) who decides to participate on the behalf of the cluster [41]. In this work, the authors noted that guardians, like physicians in conventional trials, might have potential conflicts of interest and/or scientific interests in the results. In our specific case, the guardian is generally the head of the ICU and is in a position to volunteer. The coordinating investigator contacted each of them, and we can assume that any policy-maker or chief executive of the hospital participated in making the decision. In the same way, the consent of the patient to participate or not is not really applicable. If a patient declines participation, then we can only ensure that the discomforts assessment will not be performed. However, the multicomponent program will be implemented throughout the whole ICU.

Second, the option of parallel design may be discussed. Although the large differences in patient populations between ICUs should lead us to prefer a crossover design (between-group and within-group comparisons will also be permitted [43]) over a parallel design, crossover trials imply having no carryover effect. The carryover effect corresponds to an effect that "carries over" from one experimental condition to another [44] that could potentially distort the results obtained during the second period (the experimental condition from the first time period may impact the effectiveness of the experimental condition during the second time period). In our specific case, it was not possible to hypothesize that the implementation of the multicomponent program during the first time period (6 months) would not have an effect on the second time period. The implementation of targeted interventions in the ICU will probably lead to changes in the behavior of healthcare professionals. Therefore, the observed effects will depend on the order in which the interventions will be applied [45]. We have decided that any washout period will be sufficient to prevent this carryover effect [46, 47]. Moreover, this design allows us to assess the potential sustainability of the effect of program implementation. Assessment of sustainability is an important goal of evidence-based medicine and evidence-based practice to ensure that research findings are translated into clinical practice [48]. Effectiveness (and implementation) studies must particularly examine the local circumstances in which the programs would be applied and necessarily adapted. There are no data regarding seasonality, but the case mix of diseases may be different from a period of one year to another. Therefore, it could be hypothesized that perceived discomforts might vary according to the main diagnosis leading to admission to the ICU.

Third, the fact that the healthcare team will not be blinded should be considered in the interpretation of the final results. The implementation of a program of discomfort reduction associated with systematic assessment of patient discomforts may have a direct influence on healthcare behaviors. The caregivers could make more of an effort than usual, leading to better attention to the patient’s discomfort and the potential sources of discomfort. This phenomenon is well described and is known as the Hawthorne effect, and it may involve underestimation of the intervention effect [49, 50]. This potential Hawthorne effect was actually desired during the implementation of the program and must be considered as the first stage of the multicomponent program, but this effect also applies during the baseline assessment period. Therefore, the Hawthorne effect, if present, could induce an artificial reduction in the overall discomfort score due to the implementation of the program. Suppressing this potential bias would have required administration of IPREA by actors not in the ICU and without knowledge by the ICU team, which is impracticable.

Despite the acknowledged need to consider discomfort issues, self-reported assessment remains underutilized in clinical practice. Environmental barriers have been described to explain why self-reported measures have not been routinely implemented in clinical practice [51, 52]. Time and resources are both constraints on clinicians, whose main role is to provide patient care. The ergonomics of the questionnaire, such as shorter length, an electronic format, or computer-adaptive testing, may improve the acceptability [53, 54]. Although the psychometric properties and acceptability of the IPREA questionnaire are satisfactory, the development and validation of shorter or web versions should be considered to improve its implementation and use in routine clinical practice.

In conclusion, the results of this cluster-randomized controlled study are expected to confirm that a multicomponent program of discomfort reduction may be a new strategy for the care of critically ill patients.

### Trial status

At the time of first manuscript submission, the trial was currently recruiting participants. The study completion date was October 2015.

## Abbreviations

AP-HM: Assistance Publique - Hôpitaux de Marseille
AP-HP: Assistance Publique - Hôpitaux de Paris
CONSORT: Consolidated Standards of Reporting Trials
ICH: International Conference on Harmonization
ICU: intensive care unit
IPREA: Inconforts des Patients de REAnimation (Discomforts in ICU patients)
PDCA: plan-do-check-act
RCT: randomized controlled trial
